# Long-term survival with a combination of immunotherapy, anti-angiogenesis, and traditional radiotherapy in brain metastatic small cell lung cancer: a case report

**DOI:** 10.3389/fonc.2023.1209758

**Published:** 2023-10-06

**Authors:** Yan-yan Long, Jing Chen, Yue Xie, Ying Wang, Yong-zhong Wu, Ying Xv, Ke-gui Weng, Wei Zhou

**Affiliations:** ^1^Department of Radiation Oncology Center, Chongqing University Cancer Hospital and Chongqing Cancer Institute and Chongqing Cancer Hospital, Chongqing, China; ^2^First School of Clinical Medicine, Southern Medical University, Guangzhou, China

**Keywords:** anti-angiogenesis, immune checkpoint inhibitor (ICIs), radiation therapy, small cell lung cancer (SCLC), case report

## Abstract

**Purpose:**

Brain metastases (BMs) are common in Small Cell Lung Cancer (SCLC), but the prognosis is very poor. Currently, there is no standard of care on what constitutes optimal treatment, and there is no consensus regarding maintenance therapy in SCLC.

**Case description:**

We report the case of a 55-year-old man with advanced SCLC. After the initial diagnosis, he received routine chemotherapy and chest radiotherapy but developed brain metastases with 2 lesions seven months later. We used an effective combination therapy consisting of the antiangiogenic inhibitor, Anlotinib and whole-brain radiotherapy. We then administered anti-PD-L1 immunotherapy Atezolizumab in combination with Anlotinib as long-term maintenance therapy. Twelve months later, there was a progression in one of the brain metastases. The patient underwent further stereotactic radiotherapy (SRT) for the lesion. However, after four months of treatment with SRT, the lesion began to gradually grow in size. The patient underwent surgical resection of the lesion, which confirmed radioactive brain necrosis. After a full 3-year course of anti-PD-L1 therapy, the patient discontinued immunotherapy and was administered only Anlotinib as maintenance. At the time of writing up this report, the patient was alive and the overall survival reached 41 months after the onset of BM.

**Conclusion:**

This indicated a potential synergistic effect of combined immunotherapy and antiangiogenic targeted therapy with local radiotherapy in patients with BM-SCLC and can provide directions for future clinical decisions.

## Introduction

Small cell lung cancer (SCLC) is a special type of lung tumor which is highly malignant, aggressive, and prone to widespread metastasis in the early stage. About 60% to 70% of small cell lung cancer patients are diagnosed as extensive stage (ES) (1), and the five-year survival rate is less than 8% (2).Compared to other types of cancer, the incidence of brain metastases (BMs) is relatively high. It has been reported that the incidence is about 10% of at diagnosis but may be more than 50% of 2-year survivors (3–5). Once brain metastasis occurs, it can significantly affect the general performance status (PS) and prognosis of patients (6).

Radiotherapy and chemotherapy have traditionally been the cornerstone of brain metastatic small cell lung cancer. In recent years, immunotherapy based on immune checkpoint inhibitors (ICIs), especially the antibodies targeting PD-L1 and the antiangiogenic targeted therapeutic drug, Anlotinib, was found to have a certain intracranial control rate and became the main alternative, although the overall efficacy remains moderate (7–9). In addition, the role of immunotherapy and antiangiogenic targeted therapy in the maintenance of extensive SCLC has not been recognized (10–12).

Here, we have reported the case of a patient with ES-SCLC who, even after brain metastases, was able to survive long-term with a combination of local and systemic treatments, including brain radiation, anti-PD-L1 immunotherapy, and anti-angiogenic targeting. This highlights the importance of these three combined treatment strategies. This study was approved by the Ethics Committee of the Hospital of Chongqing University Cancer Hospital, and the patient gave informed consent.

## Case description

**Figure 1:**
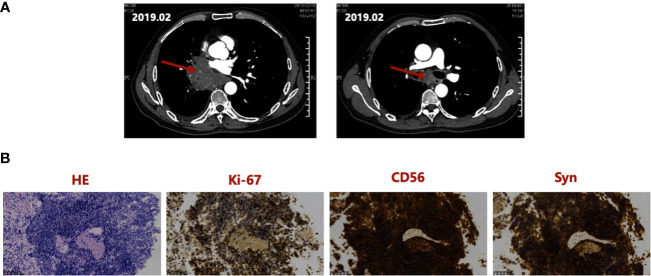
**(A)** Thoracic CT at initial diagnosis in February 2019, showing a mass of about 7.5 cm × 3.9 cm in the right central lung and the right hilar, enlarged mediastinal lymph nodes. **(B)** Hematoxylin and Eosin staining and IHC staining of the Biopsy Specimen, indicating positivity for Ki-67, CD56, and Syn. IHC, immunohistochemistry; Syn, synaptophysin.

A 55-year-old Asian male presented to a local hospital in February 2019 with a 1-week history of cough and expectoration. The Eastern Cooperative Oncology Group - Performance Status (ECOG-PS) score (13) was 1. The patient had a history of smoking for 30 years, 730 packs per year with no special medical, family, or psycho-social history. The primary physical examination showed that the right lung breathing sound of the patient was slightly weakened compared to the contralateral side, and the remaining examinations were negative. Thoracic contrast-enhanced CT (**Figure 1A**) showed a mass of approximately 7.5 cm × 3.9 cm in the center of the right lung with corresponding bronchial obstruction and obstructive inflammation encircling the vessels of the right lung, highly suggestive of bronchogenic carcinoma. At the same time, the right hilar and mediastinal lymph nodes were enlarged. Fiberoptic bronchoscopy biopsy showed middle right lung branch small cell lung cancer (**Figure 1B**). A thorough basic imaging examination, including bone ECT, brain MRI, and abdominal CT, ruled out metastatic lesions. Initial diagnosis: Small cell carcinoma of the right lung in cT4N2M0, IIIB (American Joint Committee on Cancer (AJCC) Version 8 staging criteria) (14).

The patient received two cycles of first-line intravenous chemotherapy with the EP regimen: 500 mg Etoposide (60 mg/m2, d1-5 every 21 days) +100 mg Nedaplatin (80 mg/m2, d1 every 21 days). The patient’s cough and expectoration resolved rapidly after treatment. Chest CT showed a marked reduction of the pulmonary lesion (**Supplementary Figure 1A**). However, bone ECT showed additional metastasis in the fourth thoracic vertebra (T4) (**Supplementary Figure 1B**). Given the asymptomatic bone metastasis and marked reduction of the lung lesion, he was administered a 2-cycle chemotherapy with the original EP regimen combined with the anti-PD-1 inhibitor Sintilimab (200mg) at the local hospital.

The patient came to our hospital on May 28, 2019, for follow-up diagnosis and treatment. On examination, the lesions in the pulmonary and mediastinal lymph nodes were markedly reduced and had almost disappeared (**Supplementary Figure 2A**). Based on the criteria for efficacy evaluation (Response Evaluation Criteria In Solid Tumors (RECIST) v1.1) (15) for solid tumors, we assessed the overall efficacy as partial response (PR), and we recommended chest irradiation.

**Figure 2:**
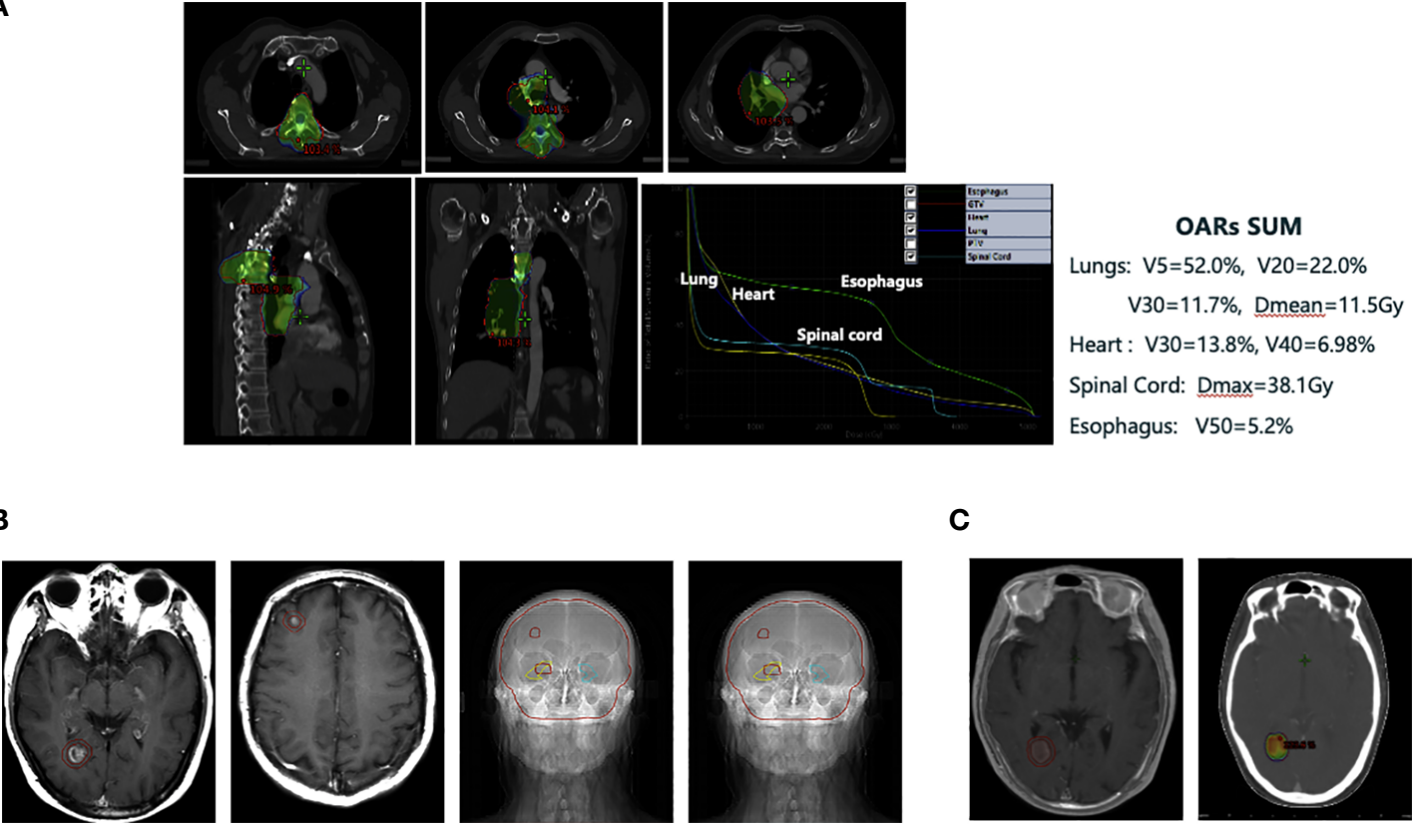
**(A)** Individualized thoracic irradiation utilizing intensity-modulated radiation therapy technique. **(B)** Whole brain irradiation (WBI) with a sequential boost to lesions, while protecting the key functional areas of the hippocampus using TOMO technique (30Gy/10F for the whole brain and the synchronous dose of 45Gy/15F for the focus). **(C)** Re-irradiation of the right occipital lobe brain metastasis with stereotactic radiotherapy (SRT) by EDGE accelerator (27Gy/3F).

From June 05 to July 10, 2019, the patient received a total dose of 36Gy/18F T4 vertebral body and 50Gy/25F thoracic involving field radiotherapy using 6MV-X-ray IMRT (**Figure 2A**). The organs at risk were within limits, well below the dose limit (for example, lung V5 = 52.0%, lung V20 = 22.0%, lung V30 = 11.7%, and lung Dmean = 11.5Gy) (**Figure 2A**). However, one month after the radiation therapy, the patient developed Grade II radiation pneumonitis according to the toxicity criteria for common adverse events (Version 4.0) (16) (**Supplementary Figure 2B**). The patient’s symptoms were significantly alleviated after oral administration of prednisone acetate tablets and other treatments. There was no subsequent recurrence of radiation pneumonitis and no hormone-related complications.

Two months after the chest radiotherapy, on re-examination, we found stable pulmonary and regional lymph node lesions, but the brain MRI (September 17, 2019) showed new enhanced nodules in the right occipital lobe (1.1 cm × 0.8 cm × 0.9 cm) and right frontal lobe (0.5 cm maximum diameter) suggesting brain metastatic lesions (**Figure 2B**). We revised the diagnosis to right lung small cell carcinoma, ycT4N2M1c, stage IVB (AJCC stage 8) with bone and brain metastases, and we evaluated the progressive disease (PD) efficacy.

At this point, the patient’s lung lesions had narrowed steadily, with two asymptomatic brain metastases, and he had recently recovered from the radiation pneumonitis. He was treated with whole-brain radiotherapy followed by targeted therapy with synchronous oral administration of Anlotinib. Whole brain irradiation (WBI) was performed using the TOMO radiotherapy technique along with a sequential boost to lesions, while protecting the key functional areas of the hippocampus (whole brain dose: 30Gy/10F, and the local dose for tumor lesions: 45Gy/15F) (**Figure 2B**). Anlotinib was prescribed as per the standards (12 mg daily orally for 14 consecutive days, repeated every 3 weeks).

After whole-brain radiotherapy, the patient showed complete recovery from radiation pneumonia (**Supplementary Figure 2C**), and immunotherapy was restarted. Anti-angiogenic targeted therapy, that is, Anlotinib combined with anti-PD-L1 Atezolizumab as maintenance therapy, was continued for a long time. There was no Anlotinib-related adverse event, such as hypertension or proteinuria, or no immune-related adverse reaction during this period, and the disease was assessed as stable with periodic imaging evaluation once every three months.

In September 2020, that is, 12 months after brain radiotherapy, the brain MRI examination showed an enlargement of the original right occipital lobe lesion (0.3cm→1.5cm) without any symptoms. After one month of follow-up, the lesion continued to grow (1.5cm→1.9cm). At that point, the extracranial lesions remained stable. Local progression of the intracranial lesions was considered clinically in combination with the medical history and with reference to MRI imaging findings. The patient refused surgery and opted for further radiation treatment of the brain lesion.

**Figure 3:**
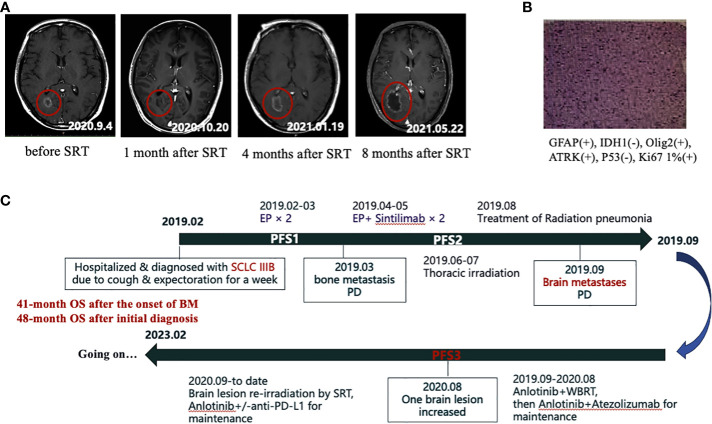
**(A)** The gradual increase of the right occipital lobe brain metastasis after SRT treatment in MRI. **(B)** Postoperative pathology of the enlarged brain lesion in the right occipital lobe, which was later confirmed as radiation necrosis. **(C)** Flow chart of the whole treatment.

From September 9 to September 11, 2020, we administered EDGE accelerator stereotactic radiotherapy (SRT) with 6 mV X-rays at a total dose of 27Gy/3F for the patient’s right occipital brain metastasis (**Figure 2C**). The metastasis was controlled for one month after SRT treatment and did not continue to grow. However, after 4 months of treatment with SRT, the aforementioned lesion began to gradually increase (**Figure 3A**). At that time, we could not completely rule out intracranial progression and considered possible radiological brain injury. On June 2, 2021, the patient underwent partial surgery for this brain lesion, which was finally confirmed as radiation necrosis (**Figure 3B**).

In October 2022, our patient stopped anti-PD-L1 immunotherapy after a full 3-year course of immunotherapy and was then continued on the single drug Anlotinib for maintenance with fine tolerance. At the last follow-up visit on 10 February 2023, overall survival (OS) from the time of first diagnosis of SCLC to present was 48 months and OS from the time of confirmation of brain metastases to present was 41 months (**Figure 3C**). To date, excellent tumor control had been achieved (**Supplementary Figure 3**) and the patient had a high quality of life.

## Discussion

Brain metastases (BMs) are common in small cell lung cancer (SCLC) and have very poor prognosis. Currently, there is no consensus on what constitutes the best treatment. Based on the IMpower133 and the CASPIAN study (7, 8), anti-PD-L1 inhibitor immunotherapy combined with chemotherapy has been recommended as a new first-line treatment for ES-SCLC. At the same time, in the ALTER 1202 study, as an anti-angiogenic tyrosine kinase inhibitor, Anlotinib was found to target VEGFR2 highly selectively, showing satisfactory intracranial control rate in brain metastatic small cell lung cancer (BM-SCLC) (9). In the treatment of ES-SCLC in third-line and above, Anlotinib improved the progression-free survival (PFS) by 3 months and the OS by 3.7 months in the brain metastasis subgroup. Therefore, as per the guidelines of the Chinese Society of Clinical Oncology (17), it has been approved for use in the treatment of ES-SCLC.

However, the efficacy of immunotherapy or antiangiogenic targeted therapy remains modest. In addition, there are mixed opinions on the maintenance therapy of ES-SCLC with no clear consensus reached (10, 11), mainly because most maintenance therapy drugs, including pembrolizumab maintenance immunotherapy, failed to show significant improvement in clinical outcomes (10). However, a randomized Phase II trial (CALGB 30504) evaluating sunitinib, a vascular endothelial growth factor receptor inhibitor, as maintenance therapy (12) demonstrated that sunitinib was safe for use in a wide range of small cell lung cancers and improved PFS (3.7 months vs.2.1 months, *P* = 0.02).

Here we have reported our individualized treatment experience consisting of a combination of systemic and local treatment strategies, including combined brain radiotherapy, anti-PD-L1 immunotherapy and anti-angiogenic targeting, and the treatment of brain metastatic SCLC(BM-SCLC) with the immune checkpoint inhibitor (ICIs) in combination with Anlotinib as long-term maintenance. The patient maintained an OS up to 41 months after the brain metastasis as of present, and the clinical efficacy was long-lasting, the patient was alive, and the quality of life was good. Now he is very satisfied with our work, and because of the good therapeutic efficacy, he is more and more cooperating with the treatment suggestions, and trusts doctors. This case proved to some extent that anti-angiogenesis can improve the efficacy of immunotherapy and prolong the time of clinical benefit.

In fact, immunotherapy combined with antiangiogenic targeted therapy is a promising strategy, which has been proved in different tumor types such as non-small cell lung cancer, hepatocellular carcinoma, and renal cell carcinoma (18). However, the evidence for this combination in small cell lung cancer remains insufficient and most are still in clinical trials (NCT04490421, NCT04192682, NCT04055792). Previous studies (18, 19) elucidated the potential mechanism of this combination therapy to improve the efficacy of immunotherapy by transforming the immunosuppressive tumor micro-environment (TME) into an immune-active TME.

In this case of BM-SCLC, local treatment was also very important, and the multidisciplinary treatment team consisting of neurosurgeons, medical oncologists, and radiation oncologists, integrated systematic and local treatments at the right time. The patient received individualized chest radiation therapy, including conventional chest involvement with adjacent T4 thoracic bone metastases, and two brain radiotherapy sessions with advanced radiation therapy techniques, including TOMO radiation to protect the hippocampus and fractionated stereotactic radiotherapy for SRT. In addition, local surgical resection was also performed when it was not possible to distinguish radiation brain necrosis from disease progression.

It has been reported that local radiotherapy (especially high-dose SRS and SBRT) had synergistic effects with anti-PD-L1 immunotherapy and anti-angiogenesis targeted therapy (20–23). Radiotherapy induces vascular normalization, enhances the release and presentation of tumor antigens, drives the infiltration of effector T cells into tumor tissue, and upregulates the expression of tumor PD-L1 and MHC-I. This upregulation can be overcome by treatment with immune checkpoint inhibitors. Anti-angiogenic therapy can promote the metastasis of immune effector cells to the tumor site to a certain extent, and limit hypoxia, thereby increasing the sensitivity of cancer cells to radiotherapy and enhancing the effect of immunotherapy. These dynamic interactions provide a theoretical basis for the combined use of immunotherapy, radiotherapy, and antiangiogenic targeted therapy for cancer management (23). This is a complex and intractable problem that needs further study.

## Conclusion

SCLC-BM remains a prominent medical challenge. In this report, we proposed an effective comprehensive treatment model combining immunotherapy, targeted therapy, and brain radiotherapy for the treatment of ES-SCLC with brain metastasis. In this case, the clinical benefit of antiangiogenic targeted therapy combined with PD-L1 inhibitors as maintenance therapy significantly inhibited the progression of extracranial tumors, with long-lasting clinical benefits. The above results highlight the importance of this comprehensive treatment strategy and can broaden therapeutic ideas, so as to provide reference for further relevant clinical research.

## Data availability statement

The original contributions presented in the study are included in the article/**Supplementary Material**. Further inquiries can be directed to the corresponding authors.

## Ethics statement

This study was conducted in accordance with the declaration of Helsinki. This study was conducted with approval from the affiliated ChongQing University Cancer Hospital (CZLS2023062A). The studies were conducted in accordance with the local legislation and institutional requirements. The participants provided their written informed consent to participate in this study. Written informed consent was obtained from the individual(s) for the publication of any potentially identifiable images or data included in this article.

## Author contributions

Conception and design of the research: Y-zW, YuX, YW, and WZ. Acquisition of data: Y-yL, JC, and YiX. Analysis and interpretation of the data: Y-zW, YuX, YW, and YiX. Statistical analysis: WZ and K-gW. Obtaining financing: Y-yL. Writing of the manuscript: Y-yL and JC. Critical revision of the manuscript for intellectual content: WZ and K-gW. All authors contributed to the article and approved the submitted version.

## Supplementary material

Supplementary Figure 1 After two cycles of intravenous chemotherapy in April 2019. **(A)** Thoracic CT shows that the lung lesion is significantly reduced. **(B)** Bone ECT indicates new metastasis of the fourth thoracic vertebra (T4).

Supplementary Figure 2 **(A)** Thoracic CT in May 2019 when the patient came to our hospital first, showing that the lesions of lung and mediastinal lymph nodes have shrunk considerably and almost disappeared. **(B)** Thoracic CT in August 2019 when one month after thoracic radiotherapy showing that the patient has Grade 2 radiation pneumonia. **(C)** Thoracic CT in June 2020, indicating that the patient completely recovered from radiation pneumonitis after treatment.

Supplementary Figure 3 Thoracic CT (left) and brain MRI (right) in October 2021. This is four months after surgery of the enlarged brain lesion in the right occipital lobe. It indicates that very good tumor control has been achieved.
