# Supplementary figures and images for: Long-term survival with a combination of immunotherapy, anti-angiogenesis, and traditional radiotherapy in brain metastatic small cell lung cancer: a case report
Source: Front Oncol. 2023 Oct 6;13:1209758. doi: 10.3389/fonc.2023.1209758 (PMC10587576; doi:10.3389/fonc.2023.1209758)

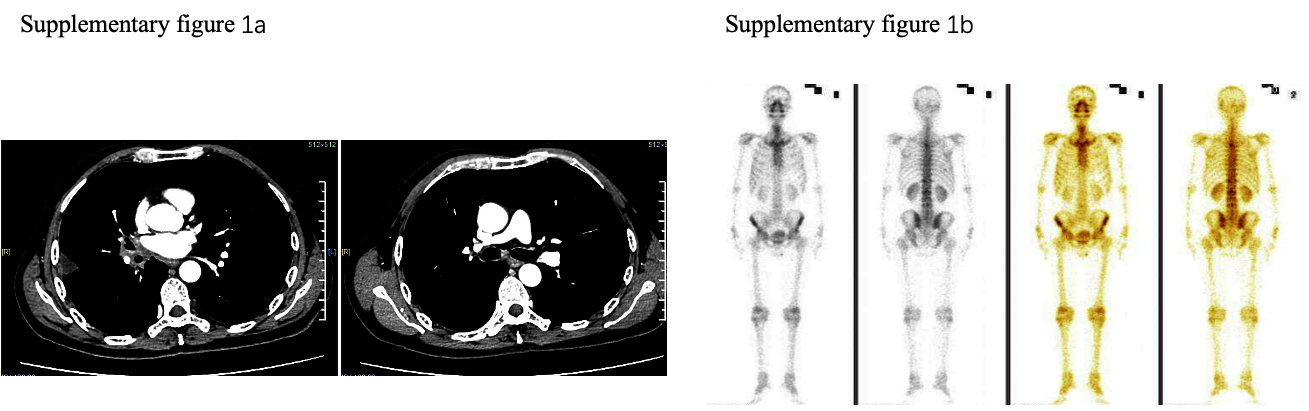

Supplement: Supplementary Figure 1 — After two cycles of intravenous chemotherapy in April 2019. (A) Thoracic CT shows that the lung lesion is significantly reduced. (B) Bone ECT indicates new metastasis of the fourth thoracic vertebra (T4). [file Image_1.tif]

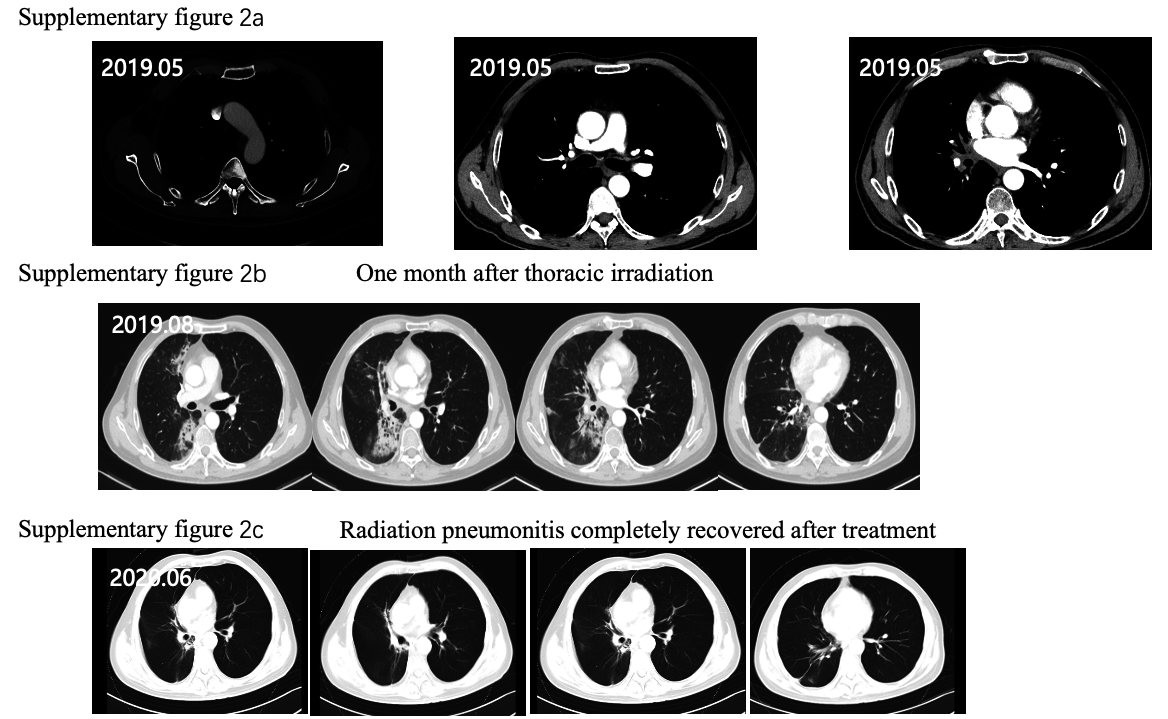

Supplement: Supplementary Figure 2 — (A) Thoracic CT in May 2019 when the patient came to our hospital first, showing that the lesions of lung and mediastinal lymph nodes have shrunk considerably and almost disappeared. (B) Thoracic CT in August 2019 when one month after thoracic radiotherapy showing that the patient has Grade 2 radiation pneumonia. (C) Thoracic CT in June 2020, indicating that the patient completely recovered from radiation pneumonitis after treatment. [file Image_2.tif]

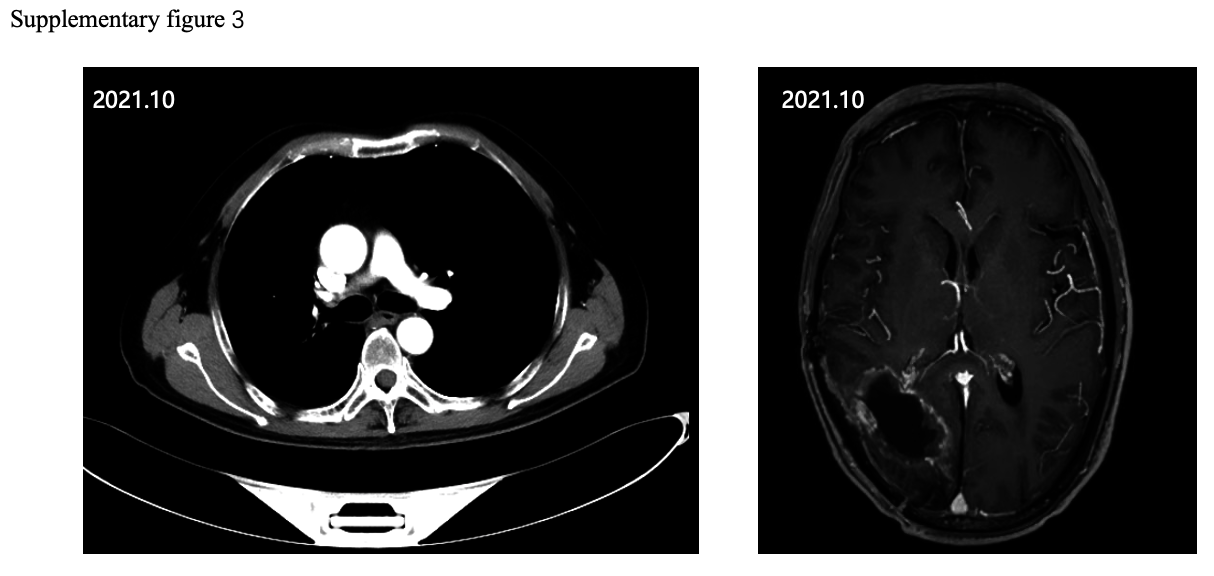

Supplement: Supplementary Figure 3 — Thoracic CT (left) and brain MRI (right) in October 2021. This is four months after surgery of the enlarged brain lesion in the right occipital lobe. It indicates that very good tumor control has been achieved. [file Image_3.tif]
